# Genetic Separation of *Listeria monocytogenes* Causing Central Nervous System Infections in Animals

**DOI:** 10.3389/fcimb.2018.00020

**Published:** 2018-02-05

**Authors:** Lisandra Aguilar-Bultet, Pamela Nicholson, Lorenz Rychener, Margaux Dreyer, Bulent Gözel, Francesco C. Origgi, Anna Oevermann, Joachim Frey, Laurent Falquet

**Affiliations:** ^1^Institute of Veterinary Bacteriology, Vetsuisse Faculty, University of Bern, Bern, Switzerland; ^2^Graduate School for Cellular and Biomedical Sciences, University of Bern, Bern, Switzerland; ^3^BUGFri Group, Swiss Institute of Bioinformatics, Fribourg, Switzerland; ^4^Division of Neurological Sciences, Department of Clinical Research and Veterinary Public Health (DCR-VPH), Vetsuisse Faculty, University of Bern, Bern, Switzerland; ^5^Centre for Fish and Wildlife Health (FIWI), Vetsuisse Faculty, University of Bern, Bern, Switzerland; ^6^Division of Biochemistry, Department of Biology, University of Fribourg, Fribourg, Switzerland

**Keywords:** *Listeria monocytogenes*, comparative genomics, virulence, lineages I and II, Reads Per Kilobase per Million Mapped, Single Nucleotide Variants, central nervous system infections, listeriosis

## Abstract

*Listeria monocytogenes* is a foodborne pathogen that causes abortion, septicemia, gastroenteritis and central nervous system (CNS) infections in ruminants and humans. *L. monocytogenes* strains mainly belong to two distinct phylogenetic groups, named lineages I and II. In general, clinical cases in humans and animals, in particular CNS infections, are caused by lineage I strains, while most of the environmental and food strains belong to lineage II. Little is known about why lineage I is more virulent than lineage II, even though various molecular factors and mechanisms associated with pathogenesis are known. In this study, we have used a variety of whole genome sequence analyses and comparative genomic tools in order to find characteristics that distinguish lineage I from lineage II strains and CNS infection strains from non-CNS strains. We analyzed 225 strains and identified single nucleotide variants between lineages I and II, as well as differences in the gene content. Using a novel approach based on Reads Per Kilobase per Million Mapped (RPKM), we identified 167 genes predominantly absent in lineage II but present in lineage I. These genes are mostly encoding for membrane-associated proteins. Additionally, we found 77 genes that are largely absent in the non-CNS associated strains, while 39 genes are especially lacking in our defined “non-clinical” group. Based on the RPKM analysis and the metadata linked to the *L. monocytogenes* strains, we identified 6 genes potentially associated with CNS cases, which include a transcriptional regulator, an ABC transporter and a non-coding RNA. Although there is not a clear separation between pathogenic and non-pathogenic strains based on phylogenetic lineages, the presence of the genes identified in our study reveals potential pathogenesis traits in ruminant *L. monocytogenes* strains. Ultimately, the differences that we have found in our study will help steer future studies in understanding the virulence mechanisms of the most pathogenic *L. monocytogenes* strains.

## Introduction

*Listeria monocytogenes* is a rod-shaped Gram-positive bacterium that is an opportunistic food-borne pathogen (Farber and Peterkin, 1991; Vazquez-Boland et al., 2001b; Conly and Johnston, 2008). It is the etiological agent of listeriosis in humans and ruminants leading to abortion, septicemia, gastroenteritis and central nervous system (CNS) infections (Low and Donachie, 1997; Oevermann et al., 2010). Based on classical molecular subtyping methods, such as ribotyping, pulse field gel electrophoresis (PFGE) and multilocus sequence typing (MLST), *L. monocytogenes* strains are grouped into four distinct phylogenetic lineages called I, II, III and IV (Ward et al., 2008; Den Bakker et al., 2010; Orsi et al., 2011). Strains belonging to lineages I and II are the most representative in terms of number of strains isolated worldwide and impact on the disease (Chenal-Francisque et al., 2011; Orsi et al., 2011). Studies based on multilocus variable-number tandem-repeat analysis (MLVA) have revealed that lineage I strains are linked to CNS infections whereas most of the food and environmental strains belong to lineage II (Balandyte et al., 2011). Likewise, it was shown in recent studies based on MLST, that lineage I is mostly composed of clinical strains, from both ruminants (Dreyer et al., 2016) and humans (Maury et al., 2016), while lineage II typically clusters food and environmental strains. Lineage III and IV strains are very rare and mostly come from animals (Kuenne et al., 2013).

*Listeria monocytogenes* is a facultative anaerobic, non-spore forming, motile intracellular pathogen that can survive and reproduce under extreme conditions. It can persist ubiquitously in the environment, such as in soil, manure and grass. It is able to proliferate inside host organisms as well as in refrigerators and food processing factories (Doumith et al., 2004). Various *L. monocytogenes* virulence factors have been identified and the most important one to date is the *Listeria* pathogenicity island number 1 (LIPI-1). This is a 9 kb long region composed of six genes encoding proteins that are required for crucial steps in the intracellular life cycle of *L. monocytogenes* (Portnoy et al., 1992; Chakraborty et al., 2000; Kreft and Vazquez-Boland, 2001; Vazquez-Boland et al., 2001a). These six genes produce important virulence factors called listeriolysin O (encoded by the *hly* gene), phospholipases A and B (encoded by *plcA* and *plcB*, respectively), zinc metalloproteinase (encoded by *mpl*), actin assembly-inducing protein (encoded by *actA)* and the transcriptional activator PrfA (encoded by *prfA)*. PrfA is a 27 kDa site-specific DNA binding protein that regulates the transcription of all LIPI-1 genes (Leimeister-Wachter et al., 1990; Vazquez-Boland et al., 2001a; Scortti et al., 2007).

PrfA also regulates virulence genes not located on LIPI-1, such as the internalin genes *inlA, inlB*, and *inlC* (Freitag et al., 2009). Internalins are a group of surface proteins that are important for the pathogenesis of *L. monocytogenes*. A large family of internalins was identified in the EGD-e strain (Bierne et al., 2007). Internalins A and B are directly associated with the invasion of the host (Gaillard et al., 1991; Dramsi et al., 1995), while internalin C is important for cell-to-cell spread (Engelbrecht et al., 1996).

Many studies concerning the molecular mechanisms of virulence in *L. monocytogenes* have been conducted using the lineage II EGD-e strain (Glaser et al., 2001), but much less research has been performed using lineage I strains, despite the fact that they typically constitute more virulent strains. To this end, we fully sequenced and assembled a lineage I strain (JF5203) that we defined as the reference of CNS infections in ruminants in order to identify virulence genes by Whole Genome Sequencing (WGS) and a comparative genomics analysis. This strain is a rhombencephalitis isolate from sequence type 1 (ST1), has been extensively characterized (Henke et al., 2015; Dreyer et al., 2016; Rupp et al., 2017). Due to its capacity to infect bovine brain cell cultures and to spread by intra-axonal migration, we have chosen this strain as it is assumed to have the molecular factors needed for neuroinvasion.

In addition to the above mentioned multilocus-based studies, large scale WGS studies recently confirmed the distribution in the four phylogenetic lineages and the clonal population structure of *L. monocytogenes* (Kwong et al., 2016; Maury et al., 2016; Moura et al., 2016). WGS approaches have also become an important tool in the epidemiological surveillance of *L. monocytogenes* (Bergholz et al., 2015; Jackson et al., 2016; Kwong et al., 2016; Maury et al., 2016). Moreover, studies analyzing large amounts of *L. monocytogenes* genomic data has led to the identification of hypervirulent and hypovirulent groups (Dreyer et al., 2016; Maury et al., 2016) and various putative virulence factors (Maury et al., 2016). Recently, a cluster of six genes called LIPI-4 and annotated as a cellobiose-family phosphotransferase system was described in clonal complex 4 (CC4) *L. monocytogenes* strains (Maury et al., 2016). LIPI-4 revealed to have CNS invasion capacity in humanized mice. However, ST1 (CC1) is devoid of LIPI-4 (Maury et al., 2016) and constitutes a predominant group in ruminant rhombencephalitis cases (Dreyer et al., 2016). Therefore, to better understand neurovirulence in *L. monocytogenes*, the aim of our study was to elucidate the characteristics that distinguish lineage I from lineage II strains in ruminants, as well as to differentiate between clinical strains (in particular CNS infection strains) from non-disease related strains, using WGS analyses and comparative genomics tools.

## Materials and methods

### Bacterial strains

A total of 121 strains from lineage I and 104 from lineage II were included in the study. These strains come from our internal collection and have been phenotypically characterized and used in previous studies (Balandyte et al., 2011; Rupp et al., 2015; Dreyer et al., 2016). The strains were isolated by enrichment in Oxoid Novel Enrichment Broth at 30°C and subsequently growth on Brilliance Listeria agar (Oxoid, Ltd., Basingstoke, United Kingdom) at 37°C for 24 h. Single colonies suggestive for *L. monocytogenes*, were then transferred to Tryptic Soy Agar (TSA) containing 5% (v/v) sheep blood (BD, Becton Dickinson and Company, Sparks, U.S.A.) and incubated at 37°C for another 24 h. For a few strains, colonies presenting haemolysis on the TSA were then applied to the VITEK Compact 2 phenotypic analysis identification system, using Gram-positive identification cards (Biomerieux, Geneva, Switzerland) for the phenotypic identification of the species *Listeria monocytogenes*. For the rest of the strains, the species and lineage were defined according to Matrix-Assisted Laser Desorption Ionization-Time Of Flight Mass Spectrometry (MALDI-TOF MS) (Dreyer et al., 2016), after confirming the equivalence between the two methods. Two strains from lineage I (JF5203 and JF5861) and two strains from lineage II (JF4839 and LMNC088) were selected as internal reference strains (Table 1). The JF5203 strain belongs to sequence type (ST) 1 and was isolated from a rhombencephalitis case in cattle. The JF5861 strain belongs to ST4 and originated from a human CNS infection. The LMNC088 strain belongs to ST412 and came from the farm environment. The JF4839 strain belongs to ST9 and originated from food not related to any listeriosis outbreak.

**Table 1:** Information about the four strains selected as internal reference strains in our study.

**Lineage**	**Strain**	**ST**	**CC**	**Source**	**Year**
Lineage I	JF5203	1	1	Cattle brain	2007
	JF5861	4	4	Human brain	2006
Lineage II	JF4839	9	9	Food (cheese)	2006
	LMNC088	412	412	Environment	2014

### Genomic DNA extraction

*Listeria monocytogenes* strains that were re-sequenced in this study were grown overnight at 37°C on TSA supplemented with 5% (v/v) sheep blood (Becton Dickinson GmbH, BD^TM^Trypticase^TM^, PA-254053.07). Colonies were picked and directly treated with lysozyme (at a final concentration of 0.4 μg/μL). Thereafter, the bacterial cells were lysed in guanidium buffer (60% w/v) (Pitcher et al., 1989) and genomic DNA (gDNA) was extracted according to a previously published phenol-chlorofom-isoamyl alcohol protocol (Wilson, 1987).

### Whole genome sequencing

Ninety-one of the total number of strains used in this study were sequenced in a previous study (Accession numbers PRJEB15123 and PRJEB15195; Dreyer et al., 2016). Some of them were re-sequenced to improve the data quality and coverage. The remaining 134 strains were sequenced specifically for this study (Table S1).

All strains were sequenced using the Illumina® technology (https://www.illumina.com/), either on MiSeq (300 bp paired-end reads) or HiSeq 2500/3000/4000 (95–150 bp paired-end reads) platforms, according to the manufacturer's protocols. Genome coverage varied from 19 x to more than 1000 x (Table S1). The four internal reference strains (JF5203, JF5861, JF4839, and LMNC088) were also sequenced using the Pacific Biosciences® (PacBio) technology (http://www.pacb.com/) in order to combine the high level of accuracy from the short-reads sequencing generated by Illumina technology with the long fragments from PacBio sequencing technology.

### Genome assembly and annotation

*De novo* assembly of PacBio data was done using HGAP v3.0 (Chin et al., 2013) from the SMRT® Analysis package v2.3.0. Quality control of the assembly was performed by mapping the Illumina reads to the obtained contigs and then performing an analysis with Qualimap v.2.2.1 (Okonechnikov et al., 2016). Circularization of the single contigs was carried out using the AMOS package v.3.1.0 (Treangen et al., 2011). Genomes were compared using the BRIG application v.0.95 (Alikhan et al., 2011) and ANI server (Rodriguez-R and Konstantinidis, 2016). Annotation of the whole genomes was made using Prokka v1.12 (Seemann, 2014) and MicroScope (Vallenet et al., 2013).

### Variant detection

To detect variations between the two lineages, Illumina reads of all the sequenced strains were mapped using BWA v0.7.13 (Li and Durbin, 2009) to the whole genome sequence of JF4839 (internal reference strain of lineage II). This was done to obtain the same position in all genomes relative to the same position in the reference genome. Reads with quality values below 20 in Sanger scale (Phred+33) were excluded from the analysis using sickle (https://github.com/najoshi/sickle). The total number of genomic variants including short insertions/deletions (INDELs) and single nucleotides variants (SNVs) were identified per strain using SAMtools v0.1.19 (Li et al., 2009). For variant calling files (vcf) filtering and manipulation SAMtools and vcflib (https://github.com/vcflib/vcflib) were used. Variants with mapping and assertion quality values lower than 30 in Phred-scale and with less than 20 reads supporting the alternate allele were filtered out from the vcf files in individual genomes. A Mann-Whitney-Wilcoxon test (“stats” R-package) was performed to identify differences between the numbers of SNVs per lineage (Neuhäuser, 2011). The vcf files per strain were then combined into a single merged vcf file using VCFtools v0.1.14 (Danecek et al., 2011). Manipulation and annotation of the merged vcf file was done with VCFtools, SnpEff v4.3i, (Cingolani et al., 2012) and *in house* bash scripts.

A phylogenetic tree based on the multiple alignment of the SNVs found (ignoring heterozygous sites) was built using RAxML v8.2.9 (Stamatakis, 2014), with a generalized time reversible (GTR) substitution model and using a strain from lineage III as an outgroup. Bootstrap scores (350 replicates) were calculated. The tree was re-rooted to the outgroup genome using FigTree v.1.4.3 (http://tree.bio.ed.ac.uk/software/figtree/), edited and displayed adding metadata information with CLC Genomics Workbench v.9.5.2 (https://www.qiagenbioinformatics.com/products/clc-genomics-workbench).

The list of variants present in lineage I with respect to lineage II was filtered by excluding the SNVs with low impact according to SnpEff (http://snpeff.sourceforge.net/SnpEff_manual.html). The same approach as detailed above for read mapping and SNVs filtering was repeated with JF5203 (lineage I, CC1 and ST1) as the reference to identify SNVs only present in lineage II strains with respect to lineage I strains.

Using an *in house* python script the variants per gene were counted in the CNS-related strains, in order to look for genes with multiple SNVs. The BED file of the annotated JF4839 genome and a filtered merged vcf file containing the variants private to CNS cases (according to SnpSift) were used as input files.

### Core- pan- genome analyses based on a reduced set of strains

Thirty-six published genomes with sufficient information about their lineages (18 belonging to lineage I and 18 to lineage II), along with the genomes of the four internal reference strains underwent pan-genome analyses (Table S2). Using the MicroScope platform (Vallenet et al., 2013), the core-genome of lineage I excluding the pan-genome of lineage II was calculated (at the protein level) for 80% sequence identity and 80% length coverage. As a result, the protein-coding genes shared by the 20 strains of lineage I but absent in any of the 20 strains of lineage II were predicted. A putative function was assigned to proteins with no described function according to InterPro (Apweiler et al., 2000) and BLASTp search (Altschul et al., 1990) against UniProtKB (Bairoch et al., 2005). The list was further filtered by taking into account the presence of certain amino acid motifs and domains potentially related to surface proteins and virulence factors (Bierne and Cossart, 2007).

### Reads per kilobase per million analysis

To check for the presence of the previously (section Core- pan- genome analyses based on a reduced set of strains) selected genes present in all lineage I strains of our set but absent in all lineage II strains, we used the information recorded in the bam files (all Illumina reads of each strain mapped to the JF5203 genome) to calculate the Reads Per Kilobase per Million mapped (RPKM) values. RPKM analysis is an established method for RNA sequencing (RNA-seq) data examination (Mortazavi et al., 2008; Deng et al., 2012; Tonner et al., 2012). It allows the RNA-seq gene expression quantification by normalizing for total read length and the number of sequencing reads. RPKM values were obtained according to the following equation:

$$\begin{array}{l} RPKM=\;\frac{numReads}{\frac{geneLength}{1000}*\frac{totalNumReads}{1000000}} \end{array}$$

*numReads* is the number of reads mapped to a gene sequence, *geneLength* is the length of the gene sequence (in bases) and *totalNumReads* is the total number of reads mapped to the genome.

Two housekeeping genes, *dnaA* and *gyrB* genes were used as controls. The Mann-Whitney-Wilcoxon test (“stats” R-package) was used to check for differences between the RPKM values for each of the previously identified genes in the two lineages (Neuhäuser, 2011).

To corroborate that there were differences between the RPKM values only in the selected genes with respect to the controls, a pairwise comparison between elements of different lineages by calculating the RPKM-difference values was performed (subtracting each RPKM value of lineage II to each RPKM value of lineage I). The post hoc Dunn's test for the Kruskal-Wallis multiple comparison test was performed with “dunn.test” R-package v.1.3.4 to assess the significance among the groups (Dinno, 2017).

### RPKM analysis at whole genome level

RPKM values were calculated in the 121 sequenced lineage I genomes and in the 104 sequenced lineage II genomes using the 2981 genes of the annotated JF5203 genome as references. Thereafter a similar procedure as described in section Reads Per Kilobase per Million analysis was performed to calculate the RPKM-difference values amongst the different lineages for all genes. The median of the RPKM-difference per gene was calculated and genes with a median greater than or equal to 233.77 (2^*^standard deviation) were retained to obtain a list of genes predominantly absent in lineage II. A matrix was built based on the RPKM values of the selected genes. A heatmap was calculated and plotted in R (“gplots” R-package) (Warnes et al., 2016) using hierarchical clustering algorithms (average linkage clustering) based on Euclidean distance.

The same RPKM approach described above for the 2981 genes in the genome was used for comparing the CNS related strains with the non-CNS infection associated ones. Genes with a median greater than or equal to 103.38 were kept. These genes are predominantly absent in food, environmental and non-neurolisteriosis strains.

Finally, the method was applied to compare strains from the clinical group (AD) with the ones present in the non-clinical group BC (Section RPKM analyses at whole genome level and PCA analysis). Genes with a median greater than or equal to 154.01 were kept.

After every comparative analysis, the selected genes underwent a Gene Ontology (GO) enrichment step by Blast2GO (Conesa et al., 2005) and Interproscan v.5.2 (Jones et al., 2014).

### Principal component analysis

A principal component analysis (PCA) of the RPKM values for the 2981 genes in the 225 strains was performed (“stats” R-package). A permutation multivariate analysis of variance (PERMANOVA) test (Anderson, 2001) was used to identify significant differences between the different clusters (“vegan” R-package) (Oksanen et al., 2017).

### Statistical analyses

All the statistical analyses were done in R v3.3.2 (R Development Core Team, 2016) and all *p*-values < 0.0001 were considered as significant.

### Accession numbers

The four reference genomes with their annotations and the sequencing data for all the strains used in this study were submitted to the European Nucleotide Archive (ENA) under the Project number PRJEB22706 (See Tables S1, S3 for details).

## Results

### Obtaining the full genome sequence of the internal reference strains

Our first aim was to use WGS and comparative genomics to find characteristics distinguishing *L. monocytogenes* lineage I strains from lineage II strains. To this end, we selected two strains belonging to lineage I (JF5203 and JF5861) and two strains of lineage II (JF4839 and LMNC088) (Table 1) and obtained their entire genome sequences using PacBio and Illumina sequencing technologies. The one-contig assembly for each of the chosen reference strains was obtained with PacBio data and the quality control of the assemblies was performed using the short reads from Illumina. Only very few bases (less than 30) needed to be corrected. The sequences were further circularized to generate whole non-fragmented circular chromosomes. Each reference genome had a chromosome size of approximately 2.9 Mb and GC content of 38% which is in line with previously published *Listeria* genomes (Hain et al., 2006).

Additionally, episomal sequences were generated for strains JF5203 and JF4839. In strain JF5203, three low coverage contigs, named 1, 2, and 3, belonging to phages were sequenced concomitantly with the bacterial genome. In the samples sequenced by PacBio the coverage of these regions was 2.4–4.7 times below the genome coverage while, they were barely detectable in the samples processed by Illumina sequencing (Image S1). The phages identified by PHASTER (Arndt et al., 2016) are the following: in contig_1 and contig_2, two intact prophages LP-030-3 (GenBank accession number NC_024384.1) and vB_LmoS_293 (GenBank accession number NC_028929.1), respectively, while contig_3 contained an incomplete prophage. They are bacteriophages of the *Siphoviridae* family Orthocluster IV which have already been described as *L. monocytogenes* phages (Denes et al., 2014; Casey et al., 2015). Phages from this cluster are typically between 38 and 41 kb long and have GC contents of 35.5–36.6%. Indeed, the phages identified here are approximately 36, 33, and 3.5 kb in contig_1, contig_2, and contig_3, respectively and each have a GC content of between 35.3 and 37.1%. Most likely contig_2 and contig_3 are part of the same phage according to a Mauve comparison (Darling et al., 2004; data not shown). Therefore, the phages identified in JF5203 have approximately the same size as those previously reported for this cluster, as well as a similar GC content. Additional examinations to verify the contiguity of contigs_2 and 3 were not performed because it was not possible to re-isolate or re-identify the phages. Additionally, an incomplete prophage is also integrated into the chromosome of JF5203.

A 74 kbp plasmid was found in the lineage II strain JF4839, isolated from cheese unrelated to a listeriosis outbreak (Filiousis et al., 2009). The plasmid contains genes associated with metal transport and resistance to cadmium and camphor (Image S2). Cadmium is an important environmental pollutant and a potent toxicant to bacteria (Trevors et al., 1986). The metal transport and resistance genes are common in environmental strains to allow them to better adapt to the different environmental conditions. This plasmid shows 99% identity at the DNA level to the *L. monocytogenes* strain N1-011A plasmid (GenBank accession number NC_022045.1), representing approximately 79% of its length.

### Genome comparisons and annotations

When using the ANI server (http://enve-omics.ce.gatech.edu/ani/) and Nucmer (Kurtz et al., 2004) to compare the reference strains from lineage I (JF5203 and JF5861) to the previously published strain F2365 from a listeriosis outbreak in California (Mascola et al., 1988), we found more than 99.6% identity at the DNA level (Image S3). On the other hand, a comparison between the reference strains from lineage II (JF4839 and LMNC088) to the EGD-e strain, showed more variation, resulting in 99% sequence identity (Image S4). The differences between the reference strains of lineage I and II were much larger representing 5.7% (Image S5).

The numeric summary of results of the annotation step using Prokka, as well as basic metrics of the genomes obtained are detailed in the Table 2. A similar number of internalin-like proteins were identified in all four reference sequences. Likewise, we examined the integrity and synteny of the LIPI-1 island in the four reference genomes and found a preserved co-localization and order of all the genes in this region (Image S6).

**Table 2:** Genes specific to lineage I selected after the RPKM comparison between the two lineages.

**Locus tag**	**Blast2GO Description**	**Length**	**Additional annotation**
LMJF5203_00387	GNAT family acetyltraansferase	136	
LMJF5203_00388	internalin	589	
LMJF5203_00428	cell surface	407	
LMJF5203_00429	family transcriptional regulator	216	
LMJF5203_00430	macrolide transporter subunit	208	
LMJF5203_00431	macrolide ABC transporter ATP-binding	224	
LMJF5203_00432	ABC transporter permease	392	
LMJF5203_00688	Uncharacterized	223	integral component of membrane
LMJF5203_00689	Uncharacterized	61	integral component of membrane
LMJF5203_00713	cell surface	824	
LMJF5203_00714	DNA-binding	217	
LMJF5203_00715	cell surface	538	
LMJF5203_01290	Uncharacterized	120	Immunity protein 51
LMJF5203_01291	cell surface	1229	
LMJF5203_01730	family transcriptional regulator	55	
LMJF5203_01731	permease	482	
LMJF5203_01732	N-acetylmuramic acid 6-phosphate etherase	296	
LMJF5203_01733	family transcriptional regulator	283	
LMJF5203_02058	cell surface	2003	
LMJF5203_02147	family transcriptional regulator	197	
LMJF5203_02312	cell surface	1529	
LMJF5203_02537	Leucine Rich repeats (2 copies)	353	
LMJF5203_02767	cell wall anchor	489	

### Variant calling at whole genome level and phylogenetic relationship determination

**Figure 1:**
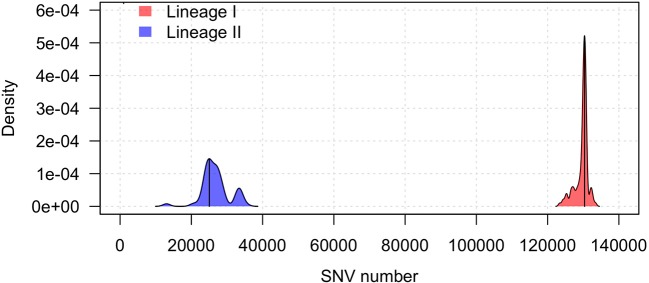
Kernel density plot of the SNV distribution per lineage taking the JF4839 strain from lineage II as a reference. Black lines represent the mode of the data. Differences between lineages were significant (*p*-value < 0.0001, Mann-Whitney-Wilcoxon test).

The Illumina sequencing data was used for the detection of variants that can distinguish between lineages I and II. Using the genome sequence of lineage II strain JF4839 as a reference to map all the strains in the study, the average number of SNVs in lineage II was 26,826 while in lineage I this value was 129,632 SNVs. The distribution of all SNVs in each lineage was represented in a kernel density plot (Figure 1, Table S4), showing more heterogeneity within lineage II (see the wider x-axis range). Significant differences with a *p*-value < 0.0001 were obtained between the two groups (Mann-Whitney-Wilcoxon test). The distribution of the total genomic variants was very similar to the distribution of the SNVs (results not shown).

**Figure 2:**
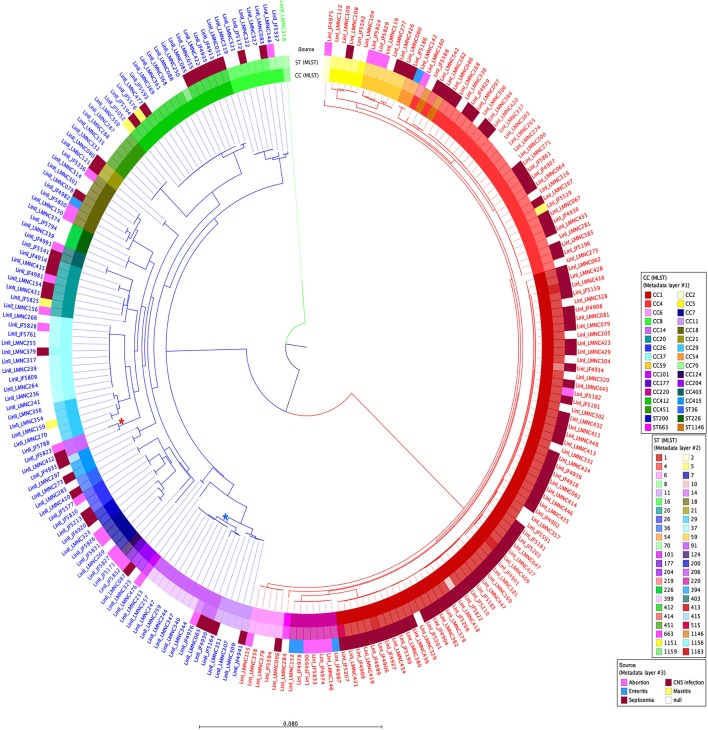
Circular dendrogram of the phylogenetic tree obtained with RAxML (Stamatakis, 2014) based on the SNVs along the whole genome of all 225 strains from lineages I and II, taking the JF4839 strain from lineage II as a reference and the LMNC318 strain from lineage III as an outgroup. Branch colors correspond to different lineages: red, lineage I; blue, lineage II; green, lineage III. Metadata is also plotted in colors as concentric rings. From inside to outside: Clonal Complex (CC) classification, Sequence Type (ST) classification, and source of infection (white color indicates food/environmental strains). ^*^Indicates the branch corresponding to ST91 (CC14), while ^*^highlights the branch where the rest of CC14 strains are located. See Table S1 for details in CC, ST and source of infection. Distance bar represents the number of substitutions per site.

A tree based on the number of SNVs at the whole genome level using JF4839 (lineage II) as reference is shown in Figure 2. A more distant strain, LMNC318 from lineage III, was used as an outgroup. In the resulting tree, three main branches are observed, corresponding to the different lineages. The topology of the tree confirms the clustering based on CC and ST classifications; interestingly a single branch of two strains corresponding to ST91 (CC14) are not grouped with other CC14 strains (see asterisks in Figure 2).

In lineage I, there are more clinical cases associated with CNS infections (75 compared to 30 cases in lineage II). One septicemia cases is also present in this group. In contrast, in lineage II, environmental strains are more common along with two strains of food origin. Other clinical manifestations such as mastitis, gastroenteritis and abortion are present in both groups. It is also relevant that lineage I has far less SNVs among them than lineage II, showing a closer distribution, while lineage II displays more diversity.

The total list of conserved variants in lineage I (present in ≥80% of the strains taking the JF4839 annotated genome of lineage II as a reference) allows the differentiation between the two lineages and all are documented in Supplementary Material DS1.

Addressing our next aim, the SNVs private to CNS infection cases were analyzed separately in order to find a pattern specific for strains of CNS origin. We looked for genes with high number of SNVs in this fraction of strains. However, we did not identify specific genes correlating to more variations in the neurolisteriosis strains. The maximum percentage of strains having common SNVs was approximately 20% (Supplementary Material DS2). Given this low percentage we think that the SNVs identified in our study are not related to neurovirulence in ruminants.

The SNVs analysis (calculation of number of SNVs per strain) was done in both directions because both lineages contain elements or genes not present in the other one. Thus, taking the JF5203 annotated genome (lineage I) as a reference, the SNVs were identified for lineage II strains. A list of variants of lineage II present in ≥80% of the strains with respect to lineage I was also created (Supplementary Material DS3).

### Differences in the thermosensor region between lineages I and II

Our whole genome variant analysis revealed variations in the *prfA* gene between lineage I and II. The PrfA protein is a master regulator essential for the activation of the transcription of many bacterial virulence factors within infected host cells. Specifically, we identified the presence of two substitutions of cytosine (C) in lineage II to thymine (T) in lineage I at positions 10 and 13 in the 5′UTR of the *prfA* gene in all of the 121 lineage I strains analyzed (Table S5). This specific untranslated region acts like a thermosensor in *L. monocytogenes* (Johansson et al., 2002).

Another pair of variants between the two lineages was found in the S-adenosylmethionine (SAM) riboswitch SreA. An Adenine (A) at position 83 in lineage II is substituted by a guanine (G) in lineage I, and a G at position 88 in lineage II is changed to an A in lineage I (Table S5). This SAM riboswitch participates in the negative regulation of PrfA translation, since it can bind and make and hybrid structure with the *prfA* transcript (Loh et al., 2009).

These two pairs of variants are given as interesting examples, however there are thousands of other potentially interesting variants to look at.

### Differential core-genome analysis between lineage I and lineage II

**Figure 3:**
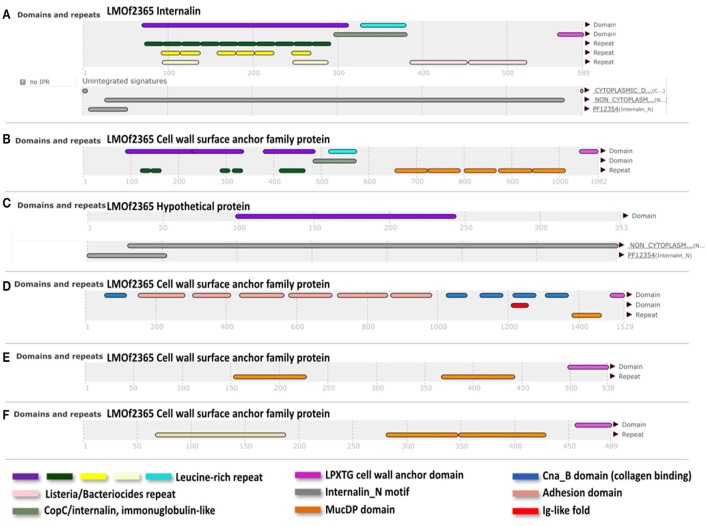
InterPro (Apweiler et al., 2000) results of the translated sequences for the six selected genes. **(A)**, Internalin (LMOF2365_RS01905); **(B)**, (LMOF2365_RS06250); **(D)**, (LMOF2365_RS11140); **(E)**, (LMOF2365_RS03470), and **(F)**, (LMOF2365_RS13380); Cell wall surface anchor family proteins. **(C)** Hypothetical protein. Nomenclature of gene products was taken from the strain F2365 annotation (lineage I).

Genetic differences between the lineages I and II are not only due to single nucleotide differences, but also due a different gene composition. Therefore, in order to identify a group of genes shared by lineage I strains but not present in lineage II, *L. monocytogenes* genomes (20 of each lineage including our references strains) were analyzed in the MicroScope platform (http://www.genoscope.cns.fr/agc/microscope/home/). The lineage I differential pan-genome comprised a total of 5,730 genes (2,838 families according to MicroScope MICFAM parameters 80% amino acid identity and 80% alignment coverage) including the lineage I differential variable-genome of 5,290 genes (2,816 families) and the lineage I differential core-genome of 440 genes. This core-genome corresponds to 22 gene families specific to lineage I (Image S7). From these 22, a further filtering was done, taking into account the gene length (longer than 90 bp, according to Prodigal Hyatt et al., 2010) and the presence of certain domains/motifs (internalin-like domains, cell wall anchor protein domains, adhesion domains such as LPXTG). According to these criteria, we identified a reduced group of six genes specific for lineage I strains (LMOF2365_RS01905, LMOF2365_RS06250, LMOF2365_RS12245, LMOF2365_RS11140, LMOF2365_RS03470, LMOF2365_RS13380), which have the potential to be putative virulence attributes of *L. monocytogenes* (Figure 3).

**Figure 4:**
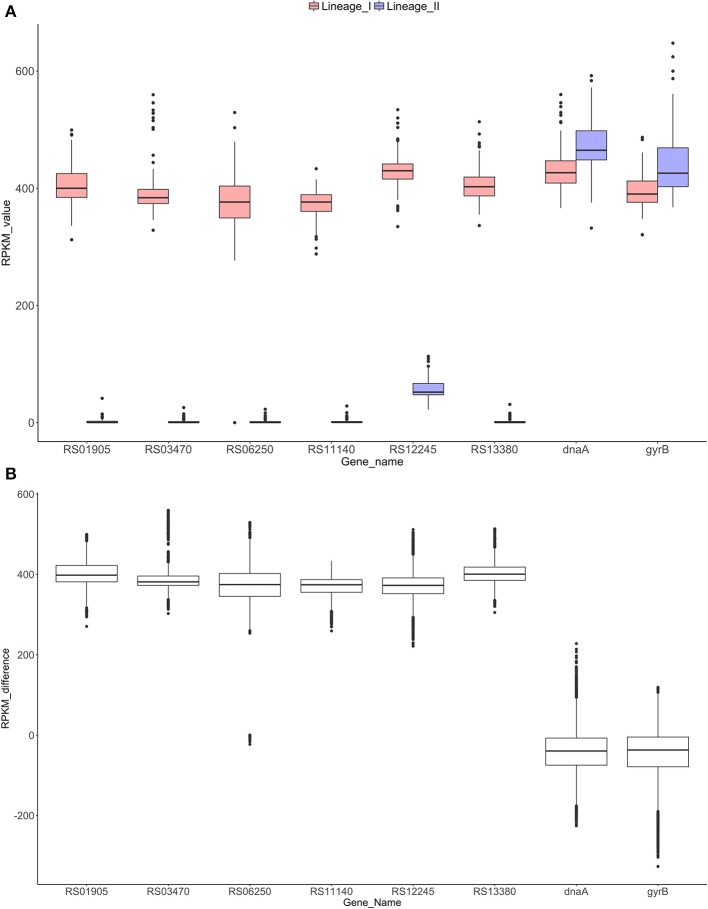
RPKM analyses to check the absence/presence of the selected genes in both lineages **(A)**. Distribution of the RPKM values in both lineages for the six selected genes and the control genes *dnaA* and *gyrB*. All the differences were significant for a *p*-value of 0.0001 (Mann-Whitney-Wilcoxon test). Gene RS12245 is affected by an artifact when counting reads in lineage II due to border effect (data not shown). **(B)** Distribution of the RPKM-differences between lineages I and II for each gene. Significant differences were found among the control groups and the six genes (*p*-value < 0.0001, Dunn's test).

In order to confirm the absence of these six selected genes in all the strains of lineage II and their exclusive presence in lineage I, we examined the remaining 221 sequenced strains. Specifically, RPKM values were calculated and compared between lineages (Figure 4A). Significant differences (*p*-value < 0.0001) were found between the lineages (Mann-Whitney-Wilcoxon test). All six genes were absent in lineage II and present in all lineage I strains analyzed, with the exception of the LMNC284 strain, where the gene LMOF2365_RS06250 is not present after verification with the Integrative Genomics Viewer (IGV) (Robinson et al., 2011; Thorvaldsdottir et al., 2013) (Image S8).

After RPKM pairwise analysis, box plots of the difference between lineage I and II gene by gene were generated (Figure 4B). For the control genes (*dnaA* and *gyrB*), the difference between the two lineages is close to zero whereas for the six selected genes, this difference increases to approximately 400, showing similar behavior for all the genes. A Dunn's test revealed significant differences (*p*-value < 0.0001) between the control group and the six selected genes.

### RPKM analyses at whole genome level and PCA analysis

The RPKM values-based analysis was extended to a whole genome level in order to explore the presence of the 2981 genes from the reference genome JF5203 in the 225 genomes of this study.

**Figure 5:**
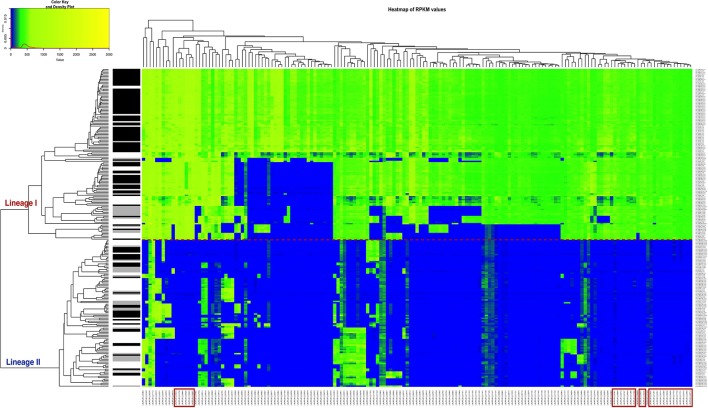
Heatmap of the RPKM values per gene for the 167 selected genes in the 225 sequenced strains after the comparison between the two lineages. Red line: separation between the lineages I and II. Gray scale color bar corresponds with the source of infection: black, CNS infection strains and one septicemia case; gray, other clinical manifestations; white, environmental and two food strains. Red boxes denote genes that are specific to lineage I.

A heatmap showing the degree of presence/absence of the 167 genes predominantly absent in lineage II was created for the 225 strains (Figure 5, Table S6). For the gene filtering, a cut-off of two times the standard deviation of the RPKM-difference values was selected (Section RPKM analysis at whole genome level). In Figure 5, the heatmap shows that the two lineages are perfectly separated based on the genes selected and that the strains generally grouped according to the CC classification, except for the same two strains of ST91 (CC14) already mentioned in section Variant calling at whole genome level and phylogenetic relationship determination (Data not shown in the Figure 5; refer to Table S1 for CC/ST classification). Furthermore, it was possible to distinguish 28 genes that are only present in lineage I (Table 2). After Blast2GO analysis, only 5 proteins remain uncharacterized, while the remaining 23 are proteins of interest because of their classification (internalin-like proteins, cell wall anchor proteins, transcriptional regulators, ABC transporters). Notably, the majority of them are bacterial surface proteins.

**Figure 6:**
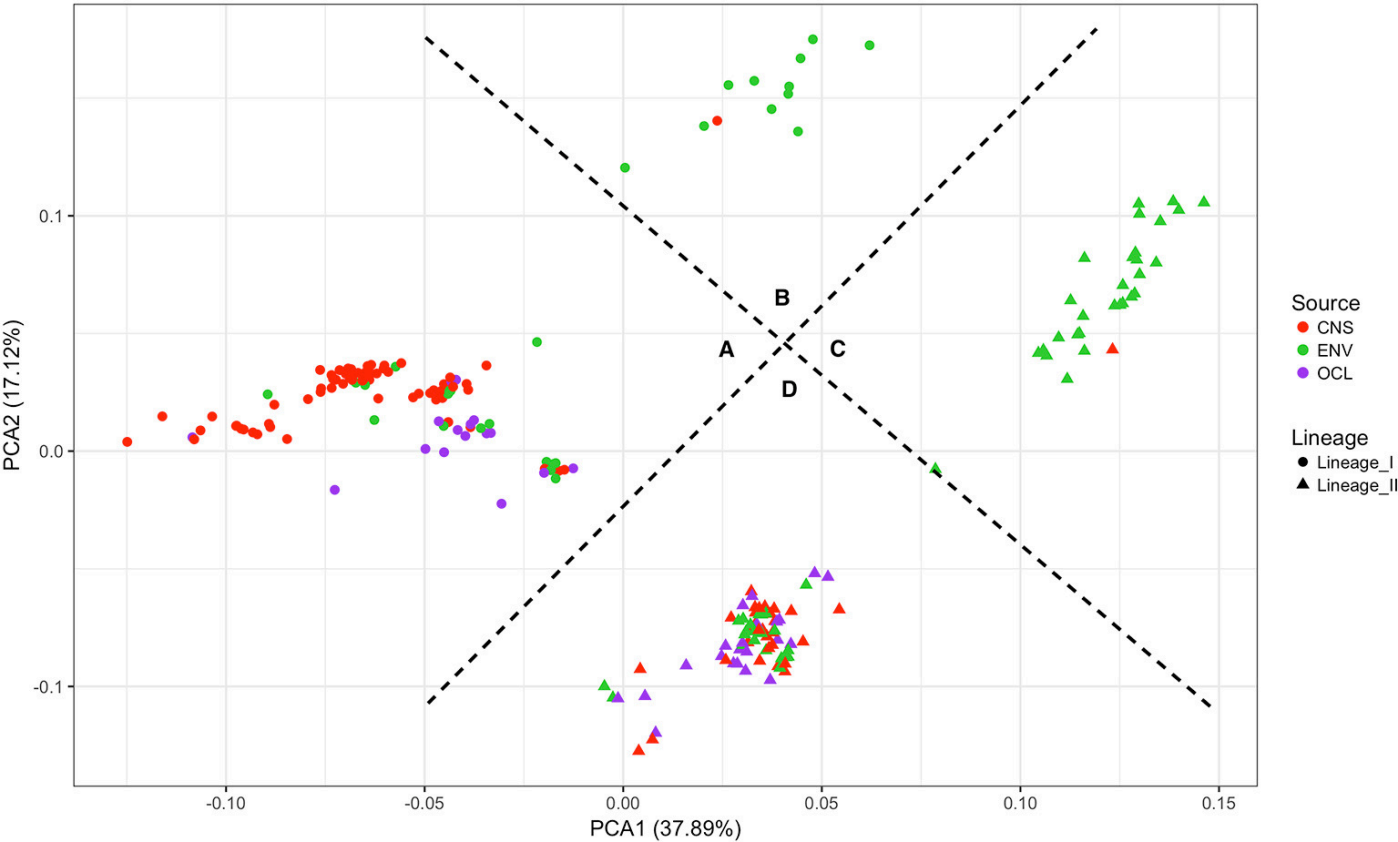
Principal component analysis of the RPKM matrix. Four groups are defined based on the clustering. **(A)** lineage I clinical, **(B)** lineage I non-clinical, **(C)** lineage II non-clinical and **(D)** lineage II clinical.

Figure 6 shows the results of a PCA analysis with the whole RPKM matrix (RPKM values of all 2981 genes in all 225 strains). PCA 1 and 2 explains the 55% of the variance. Four groups are clearly defined. On one side the lineages are perfectly separated with significant differences (*p*-value < 0.0001; PERMANOVA test). On the other side, two other groups can be distinguished, one with the majority of the clinical strains (either from lineage I or II) and the other one with the majority of the environmental strains. Significant differences were also detected with the PERMANOVA test (*p*-value < 0.0001). Based on these results, the groups were defined as follows: A-lineage I clinical, B-lineage I non-clinical, C-lineage II non-clinical, and D-lineage II clinical. Only one strain (JF5593) was not classified as either clinical or non-clinical, since is located at the middle of the two groups.

**Figure 7:**
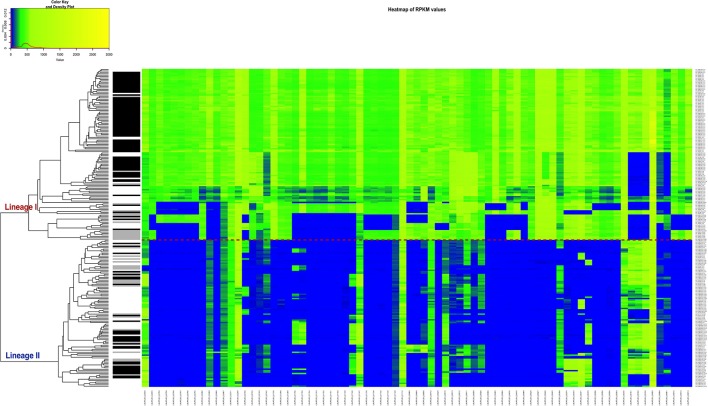
Heatmap of the RPKM values per gene for the 77 selected genes in the 225 sequenced strains after the comparison between the CNS-infection strains and non-CNS infection strains. Red line: separation between the lineages I and II. Gray scale color bar corresponds with the source of infection: black, CNS infection isolates and one septicemia case; gray, other clinical manifestations; white, environmental and two food strains.

In order to apply a clinically relevant filter to look for genes that could possibly be related to CNS infections, the RPKM method was applied, but this time to compute the differences of RPKM values between the CNS infection-related and non-related strains. We found that 77 genes are predominantly absent in the non-CNS group of the strains. According to our data, a single gene cannot perfectly separate the two groups and be assigned as CNS-infection causative, but the combination of various genes could be a signature of neurolisteriosis (Figure 7, Table S7).

There are 65 mutual genes from the 167 predominantly absent in the less virulent lineage II and the 77 genes predominantly absent in the non-CNS group. Of these 65 genes, 24 encode for membrane proteins, 5 for transcriptional regulators and the rest have other functions or are hypothetical proteins (asterisks in Table S6). These genes would be of further interest since are differentially present in strains that are pathogenic and have been associated to a CNS-infections.

**Figure 8:**
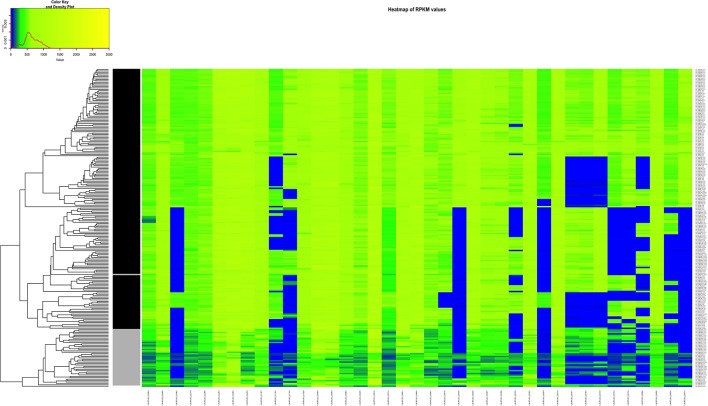
Heatmap of the RPKM values per gene for the 39 selected genes in the 225 sequenced strains after the comparison between the clinical and non-clinical group. Gray scale color bar corresponds with the classification of the groups: black, clinical group; gray, non-clinical group; white, unclassified strain JF5593.

We decided to do a comparison between the clinical AD group vs. the non-clinical BC group because of the PCA results and considering the fact that strains originating in the environment or food does not exclude the possibility of potential pathogenicity. In this case, the combination of the resulting 39 genes seem to be essential to visibly separate clinical from the non-clinical strains (Figure 8, Table S8).

Combining all the methods together and looking for common genes among the three comparisons, we have compiled a list of six genes that may represent interesting targets for future *in vitro* and *in vivo* studies to evaluate their neuroinvasion potential. These genes are: LMJF5203_02482 (transcriptional regulator), LMJF5203_01155 (ncRNA rli38) (Toledo-Arana et al., 2009), LMJF5203_00294 (ABC transporter ATPase), LMJF5203_00370 (uncharacterized conserved protein) and two short membrane proteins LMJF5203_00013 and LMJF5203_00470.

## Discussion

Recent studies using many *L. monocytogenes* strains have demonstrated that strains from lineage I are more frequently associated with clinical cases of listeriosis, both in animals or humans, while lineage II strains are more often found in food and environmental samples (Balandyte et al., 2011; Orsi et al., 2011; Dreyer et al., 2016; Maury et al., 2016). To date, there is little information concerning the molecular factors associated with CNS invasion. Therefore, the main aim of our study was to elucidate the characteristics distinguishing lineage I strains from lineage II and clinical strains from non-disease related strains, with a particular emphasis on CNS infection strains. To do this, we selected four internal references strains JF5203, JF5861, JF4839, and LMNC088 (Table 1). We used both Illumina and PacBio sequencing technologies in order to obtain complete chromosome and any additional episomal sequences.

Thereafter, we analyzed the differences between the two lineages with the goal of identifying variants and/or genes that can distinguish lineages or other phenotypic differences. The localization of these SNVs could have a potential relevance for pathogenesis. Since not all of the genes involved in virulence have been found for *L. monocytogenes*, we decided to report the list of SNVs in all genes of our reference strains: JF5203 (rhombencephalitis isolate from lineage I) and JF4839 (food isolate not related to an outbreak from lineage II). We reported the SNVs in intergenic regions and those in coding regions that have a moderate or high impact, according to SnpEff classification (Supplementary Materials DS1, DS3). Reporting the SNVs with respect to JF5203 gives a putative list of nucleotide changes that could explain pathogenesis in JF5203 (e.g., SNVs in environmental strains and not in pathogenic strains), while reporting the SNVs with respect to JF4839 offers a list of variations presumably related to loss or lack of pathogenesis in JF4839 (e.g., SNvs in pathogenic strain and not in environmental strains).

The phylogenetic tree based on multiple alignment of the SNVs along the whole genome of the reference, clearly shows three main branches corresponding to the different lineages (Figure 2). It also confirms that lineage I strains are more conserved while lineage II strains are more diverse. Overall, the clustering of the strains corresponds to the CC and ST scheme. This is also evident in the dendrogram showing the clustering based on RPKM values (Figure 5, Table S1). However, we consider that the distribution based on whole genome analysis (SNV and RPKM methods), offers more information than standard MLST classification, which is based on only 7 house-keeping genes. The possible inconsistencies between the whole genome based methods and MLST-based method may also be due to the high level of similarity of the strains, which is also indicated by the relatively low bootstrap values at many tips of the tree.

The pathogenicity island LIPI-1 is considered to be the main virulence attribute of *L. monocytogenes* and *prfA* gene is the master regulator of this island and other virulence genes (Wernars et al., 1992; Bohne et al., 1994; Scortti et al., 2007; De Las Heras et al., 2011). Temperature is important in the activation of the PrfA mRNA translation. Specifically, the 5′-untranslated region (5′UTR) of the PrfA mRNA acts like a thermosensor. At low temperatures (30°C), it forms a secondary structure in form of a hairpin that masks the ribosome binding site (RBS) hindering its translation. While, the structure melts and opens at higher temperatures (37°). This permits access to the RBS and translation of the PrfA mRNA, which is crucial for the activation of other virulence genes (Johansson et al., 2002). In the 225 strains, two characteristic differences that separates all strains between lineage I and lineage II were found, suggesting a possible role in the regulation of the virulence genes under the control of the PrfA protein potentially via thermodynamic stability. Future work, should examine if the thermodynamic structural differences of the 5′UTR of *prfA* between lineage I and II make lineage I strains intrinsically better prepared for a change from low (environmental) to higher temperatures (host), by activating the transcription of the downstream virulence genes, faster than lineage II strains.

The differences between the two lineages are not only due to the single point variants. Differential gene composition is another important criterion. Genes contained in lineage I but missing in lineage II are of particular interest because they represent a list of potential genes or regulatory elements that might facilitate the invasion of the bacteria ultimately producing CNS disorders. Several of these genes encode proteins with particular domains or motifs related to surface location, cell adhesion and internalin-like features.

Internalins are a family of surface proteins typically from *L. monocytogenes*. They are known virulence factors involved in the bacterial colonization and cell-to-cell spread in the host (Bierne et al., 2007). Internalins usually have different repeats or motifs, such as internalin_N (PF12354), Leucine-rich-repeats (LRR) domains, LRR adjacent domains, Ig-like fold regions, Mucin-Binding Protein (MucBP) repeats and LPXTG motifs. A combination of some of these elements was present amongst the six selected proteins exclusive to lineage I (Figure 3). According to their annotation in the F2365 genome, one of the shortlisted proteins corresponds to a hypothetical protein (LMOF2365_RS12245). In this hypothetical protein, we identified an internalin_N motif and a LRR domain. Among the remaining five proteins, one is classified as an internalin (LMOF2365_RS01905) with no further details, and the other four are cell wall surface anchor proteins (LMOF2365_RS03470, LMOF2365_RS06250, LMOF2365_RS11140, and LMOF2365_RS13380). Cell wall surface anchor proteins have been reported to be important in bacterial adherence, motility and survival within the host. They are also involved in the controlled synthesis and turnover of peptidoglycan (Navarre and Schneewind, 1999). Three of these proteins (LMOF2365_RS01905, LMOF2365_RS06250 and LMOF2365_RS12245) were identified before as being specific to lineage I (Bierne et al., 2007), but taking only into account three strains of lineage I (F2365, H7858, and Clip80459) and two strains of lineage II (EGD-e and F6854).

Upon analysis of the read mapping to the six gene sequences in the 225 strains, we could show that they are absent in lineage II and present in lineage I. In general, surface proteins are very important for the pathogen, as they constitute the first point of contact of the bacteria with the host, and in many cases, an effective infection process depends on them. Hence, the six genes found in lineage I but not in lineage II strains are potential candidates to play a role in pathogenicity.

For the comparative genomics study, we successfully used the Microscope platform in a first step with 36 published genomes and our 4 references strains. MicroScope constitutes a user-friendly web-based framework with several integrated tools for analyzing individual or groups of genomes. However, this system requires the assembled genomes to be previously uploaded in the platform. The submission process can take from 4 to 10 weeks per batch and only ten genomes can be compiled per batch (http://www.genoscope.cns.fr/agc/microscope/home/).

All widely used core-pan genome methods require a preliminary assembly of the reads and allele calling (e.g., PGAP Zhao et al., 2012, Roary Page et al., 2015, BPGA Chaudhari et al., 2016, panX Ding et al., 2018, etc.). For the comparative genomic analysis of the 225 *L. monocytogenes* strains against one reference strain, we propose to use directly the raw sequencing data in a novel targeted approach based on RPKM values calculation This method is less strict than the pan- core-genome performed with MicroScope or other similar methods. The mapped reads to the selected reference can be directly used to solve questions. The generation of high quality assemblies, in some cases, can require long calculation times and is prone to errors. The method developed here is faster because it only requires remapping the reads and calculating the RPKM values. These values provide a gradual quantification of the presence of the genes the different groups compared. We are aware of the limitations of this method; for example, we do not take into account all genes absent in our reference genome, which may include additional virulence factors potentially related to *L.monocytogenes* pathogenicity and neuroinvasion. In addition, we cannot address genes of which their absence could lead to pathogenicity.

In the RPKM analysis at the whole genome level, an array of presence/absence genes for all the strains was established with a heatmap. A set of 167 genes predominantly absent in lineage II was identified and from this list 28 genes are exclusive to lineage I (Figure 5). This list also includes the above-mentioned six genes identified by the core- pan-genome analysis, which constitutes a way of evaluating the effectiveness of our method.

The performance of this new approach was also evaluated by a PCA analysis, in which all the RPKM values for 2981 genes of the 225 strains were plotted (Figure 6). The graph evidenced that based on our data, the strains can be grouped following their lineage and also their clinical designation. Based on this graph, a new classification was assigned to the strains giving them a putative implication in pathogenesis: clinical group or non-clinical group. The disease-associated strains (clinical group) are significantly grouped together, with the exception of two small ruminant rhombencephalitis strains in the non-clinical group.

We examined some of the environmental strains that clustered together within the clinical group, and some of them are outbreak-related. Namely, the human brain strain, LMNC108, the two environmental strains LMNC104/109 and one food strain LMNC112 are most probably related to a local outbreak in Switzerland in 2005.

We have other cases in which the environmental strains were isolated in the same farm where outbreaks took place some years ago. This is the case for strain JF5591 that was found in the same farm as the clinical strain LMNC382 which was isolated one year previously. Furthermore, LMNC328/329 and 331 were isolated in a farm with reported outbreaks 5 years before, and LMNC302/304 and 305 were found in the same farm where the clinical LMNC378 strain was isolated two and a half year prior. All of this indicates the persistence of outbreak strains in ruminant farms.

Since it was not possible to clearly separate the pathogenic form the non-pathogenic strains by lineage classification, two other comparisons were performed: CNS-infection strains against non-CNS infection strains and strains belonging to the clinical group with strains form the non-clinical group (Figures 7, 8). Our results suggest that not one single gene, but a gene signature might be implicated in the increased virulence of specific *L. monocytogenes* strains. This characteristic has been described for other bacteria. For example, in the different pathotypes of *Escherichia coli*, such as enteropathogenic *E. coli* (EPEC), enterotoxigenic *E. coli* (ETEC), enterohaemorrhagic *E. coli* (EHEC), enteroaggregative *E. coli* (EAEC) and enteroinvasive *E. coli* (EIEC), it has been reported that depending on the pathotype, a group of genes are responsible of the particular pathogenesis (Kaper et al., 2004; Chapman et al., 2006). The six genes identified by RPKM in our study, that may play a role in neurolisteriosis, represent our targets for future *in vitro* and *in vivo* studies.

## Conclusions

In this study, we sequenced and fully assembled two lineage I and two lineage II *L. monocytogenes* strains and used them as reference genomes. Subsequently, we sequenced 221 additional strains of both lineages and performed whole genome comparative analyses using a variety of different approaches. We identified a list of private SNVs exclusive to each lineage. While, we could not observe a typical pattern for CNS infection associated strains based on SNVs, we did find two interesting variants in the important 5′UTR of the *prfA* gene. Future studies should examine if these variants provide an adaptive advantage for the pathogenic strains of lineage I compared to the food and environmental strains of lineage II. Based on a core- pan-genome analysis of published *L. monocytogenes* genomes together with our reference genomes, we identified a set of putative virulence proteins that are present exclusively in lineage I. Subsequently, a new method based on RPKM-difference values was developed for performing a rapid comparative genomic analysis of our hundreds of strains. After comparing all lineage I strains vs. lineage II, CNS-related strains against non-CNS strains and strains belonging to the clinical group to the ones present in the non-clinical group, a common fraction of 6 genes seems to be relevant for the increased virulence of the CNS disease-related strains. In addition, our study indicates that although there is not a well-defined separation between pathogenic and non-pathogenic strains according to their phylogenetic lineages, the existence of the genes identified suggests a better indication of pathogenesis in the ruminant *L. monocytogenes* strains analyzed. This work provides an excellent basis for future studies aiming to elucidate *L. monocytogenes* virulence mechanisms.

## Author contributions

LA-B performed all the bioinformatics and statistical analysis, contributed to the design of the study and wrote the article. PN participated in the design of the study and wrote the manuscript. LR assisted with the development of the scripts. MD contributed to the sample collection and gDNA purification. FO, BG and AO collaborated in the design of the study. JF and LF supervised and conceived the whole study, and wrote the manuscript. All the authors contributed to the article writing.

### Conflict of interest statement

The authors declare that the research was conducted in the absence of any commercial or financial relationships that could be construed as a potential conflict of interest.
