# Supplementary material for: Genetic Separation of Listeria monocytogenes Causing Central Nervous System Infections in Animals
Source: Front Cell Infect Microbiol. 2018 Feb 5;8:20. doi: 10.3389/fcimb.2018.00020 (PMC5807335; doi:10.3389/fcimb.2018.00020)
Supplement: Supplementary file 3 [file Table3.DOCX]

**Table S3.** General features of the *L. monocytogenes* genome sequences of the four reference strains JF5203, JF5861, JF4839 and LMNC088.

| Lineage | Strain | Chromosome size | GC content | Genes | CDS | tRNA | rRNA | ncRNA | ENA Accession number |
| --- | --- | --- | --- | --- | --- | --- | --- | --- | --- |
| Lineage I | **JF5203** | 2’900’890 | 38.0% | 2979 | 2797 | 68 | 18 | 96 | ERS1939667 (SAMEA104314689) |
|  | **JF5861** | 2’913’714 | 38.1% | 2994 | 2812 | 68 | 18 | 96 | ERS1939668 (SAMEA104314690) |
| Lineage II | **JF4839** | 2’961’681 | 38.0% | 3199 | 3013 | 68 | 18 | 100 | ERS1939665 (SAMEA104314687) |
|  | **LMNC088** | 2’935’991 | 38.0% | 3044 | 2858 | 69 | 18 | 99 | ERS1939669 (SAMEA104314691) |
