# Supplementary material for: Genetic Separation of Listeria monocytogenes Causing Central Nervous System Infections in Animals
Source: Front Cell Infect Microbiol. 2018 Feb 5;8:20. doi: 10.3389/fcimb.2018.00020 (PMC5807335; doi:10.3389/fcimb.2018.00020)
Supplement: Supplementary file 5 [file Table5.DOCX]

**Table S5.** Single nucleotides variants found in two regulatory elements in Listeria monocytogenes, 5’UTR of *prfA* gene and SAM riboswitch *sreA*.

| Gene name | Nucleotide in lineage I | Nucleotide in lineage II | Position in the JF4839 genome | Position in the gene (citation) |
| --- | --- | --- | --- | --- |
| 5’UTR *prfA* | T | C | 204’456 | 10 (Johansson et al., 2002) |
|  | T | C | 204’453 | 13 (Johansson et al., 2002) |
| *sreA* | G | A | 2’508’292 | 83 (Loh et al., 2009) |
|  | A | G | 2’508’287 | 88 (Loh et al., 2009) |
