# Supplementary material for: Genetic Separation of Listeria monocytogenes Causing Central Nervous System Infections in Animals
Source: Front Cell Infect Microbiol. 2018 Feb 5;8:20. doi: 10.3389/fcimb.2018.00020 (PMC5807335; doi:10.3389/fcimb.2018.00020)
Supplement: Supplementary file 9 [file Image1.PDF]

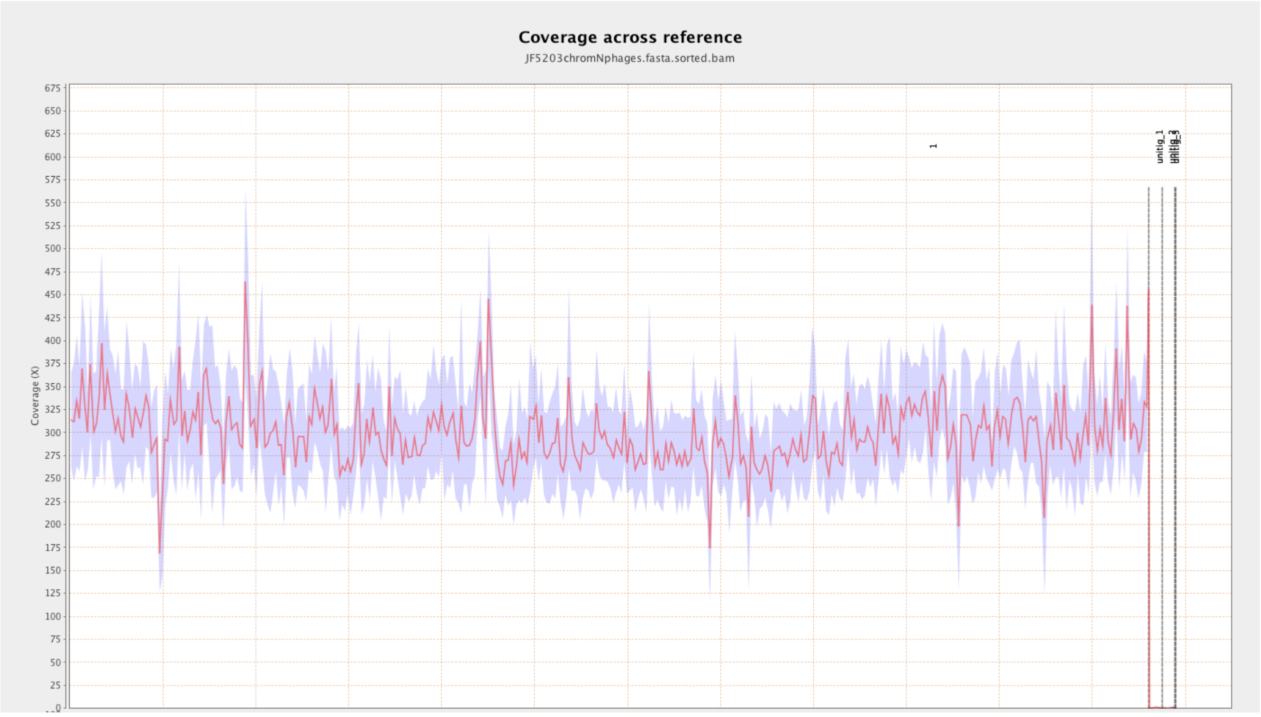

Image S1A.

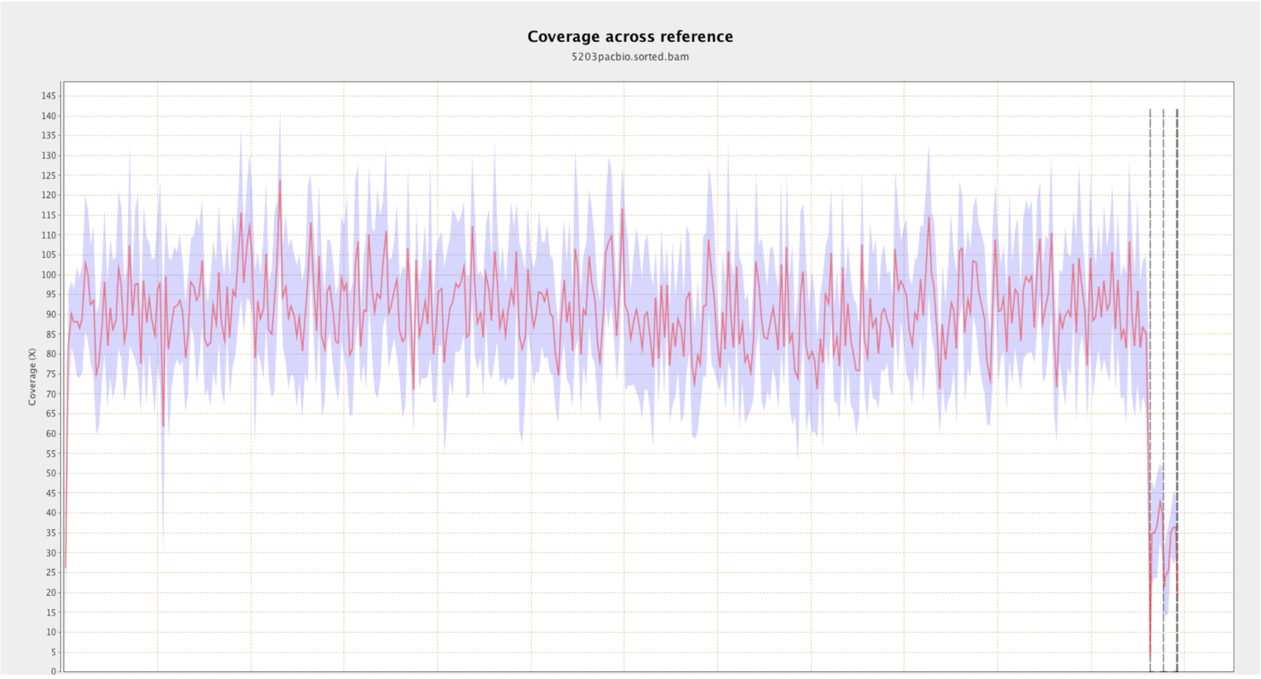

Image S1B.

Image S1: Genome coverage plot of the JF5203 strain done with the Qualimap software (Okonechnikov et al., 2016). Red lines represent the mean of the coverage. **A:** Illumina sequencing data. **B:** PacBio sequencing data.
