# Supplementary material for: Genetic Separation of Listeria monocytogenes Causing Central Nervous System Infections in Animals
Source: Front Cell Infect Microbiol. 2018 Feb 5;8:20. doi: 10.3389/fcimb.2018.00020 (PMC5807335; doi:10.3389/fcimb.2018.00020)
Supplement: Supplementary file 10 [file Image2.PDF]

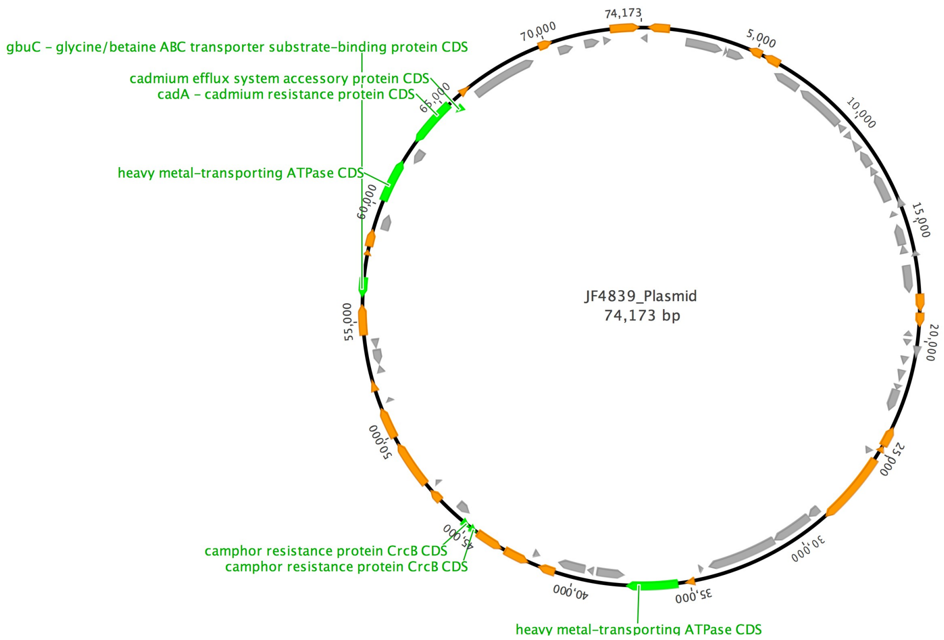

Image S2: Diagram of the plasmid found in JF4839 done with Geneious program (http://www.geneious.com). In green: genes related to metal transport, cadmium and camphor resistance and osmoregulation. In grey: hypothetical proteins CDS. In yellow: rest of the genes.
