# Supplementary material for: Genetic Separation of Listeria monocytogenes Causing Central Nervous System Infections in Animals
Source: Front Cell Infect Microbiol. 2018 Feb 5;8:20. doi: 10.3389/fcimb.2018.00020 (PMC5807335; doi:10.3389/fcimb.2018.00020)
Supplement: Supplementary file 12 [file Image4.PDF]

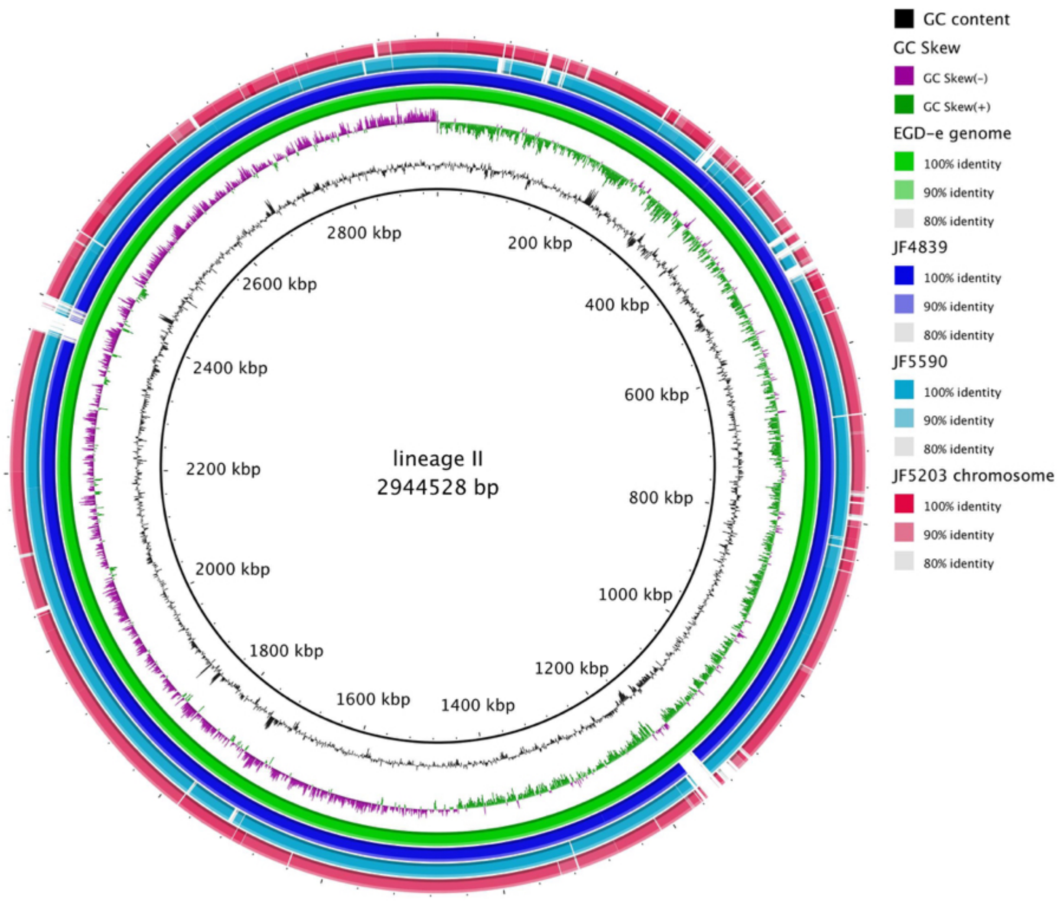

Image S4: Circular representation of the comparison between *Listeria monocytogenes* chromosomes generated with the BRIG program (Alikhan et al., 2011). From inside to outside: genomic coordinates of the reference genome in kb, GC content, GC Skew, genomes sequences. Lineage II strains taking the EGD-e genome as a reference.
