# Supplementary material for: Genetic Separation of Listeria monocytogenes Causing Central Nervous System Infections in Animals
Source: Front Cell Infect Microbiol. 2018 Feb 5;8:20. doi: 10.3389/fcimb.2018.00020 (PMC5807335; doi:10.3389/fcimb.2018.00020)
Supplement: Supplementary file 14 [file Image6.PDF]

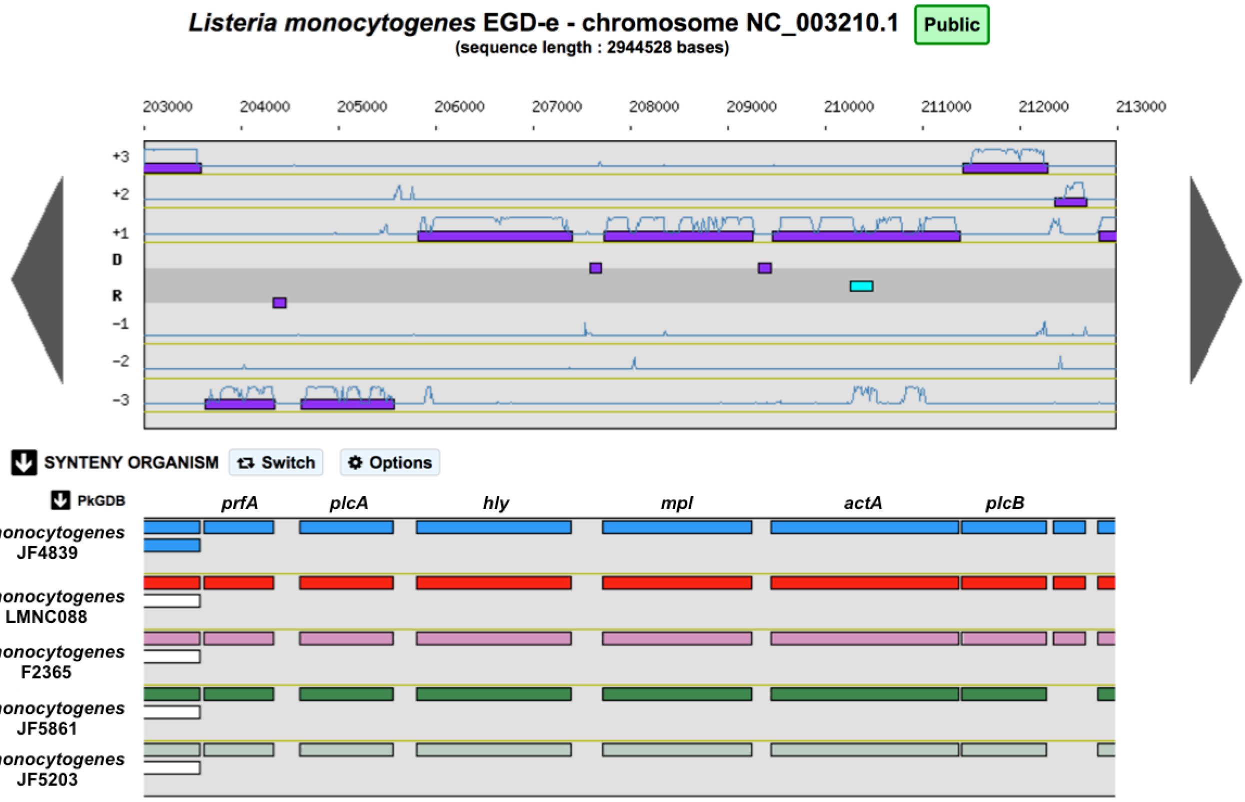

Image S6. Synteny graph of the LIPI-1 region in the four internal reference genomes taking *Listeria monocytogenes* EGD-e strain as a reference, according to MicroScope platform (Vallenet et al., 2013).
