# Supplementary material for: Genetic Separation of Listeria monocytogenes Causing Central Nervous System Infections in Animals
Source: Front Cell Infect Microbiol. 2018 Feb 5;8:20. doi: 10.3389/fcimb.2018.00020 (PMC5807335; doi:10.3389/fcimb.2018.00020)
Supplement: Supplementary file 15 [file Image7.pdf]

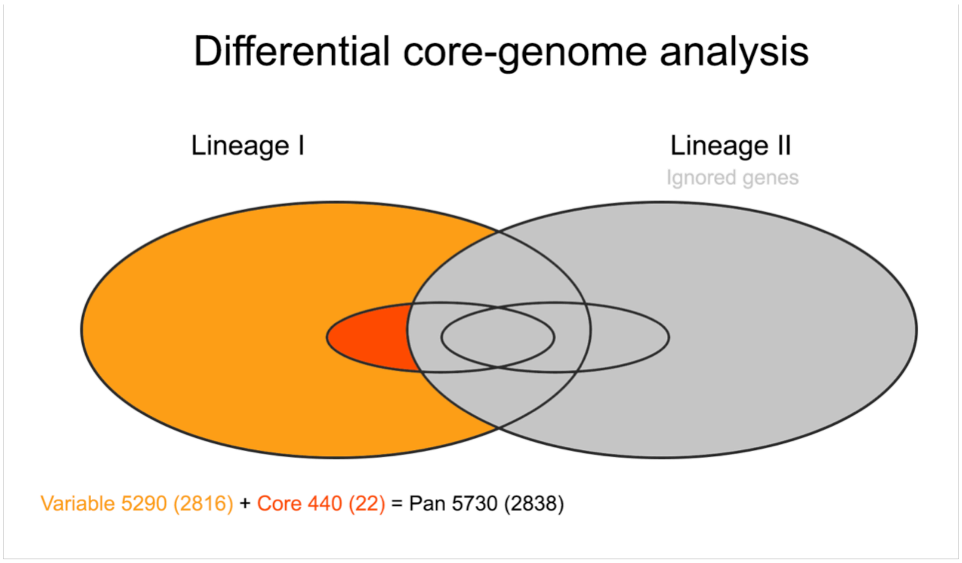

Image S7. Venn diagram representing the differential core-genome analysis. In this figure, each lineage pan genome (all genes of one lineage) is represented by the outer ring, and each lineage core genome (all core genes of one lineage) by the inner ring. The genes that are greyed out are ignored because they are found in lineage II. The orange genes represent the differential accessory or variable genome of lineage I (not present in lineage II). The red genes represent the differential core-genome of lineage I not present in lineage II.
