# Supplementary material for: Genetic Separation of Listeria monocytogenes Causing Central Nervous System Infections in Animals
Source: Front Cell Infect Microbiol. 2018 Feb 5;8:20. doi: 10.3389/fcimb.2018.00020 (PMC5807335; doi:10.3389/fcimb.2018.00020)
Supplement: Supplementary file 16 [file Image8.PDF]

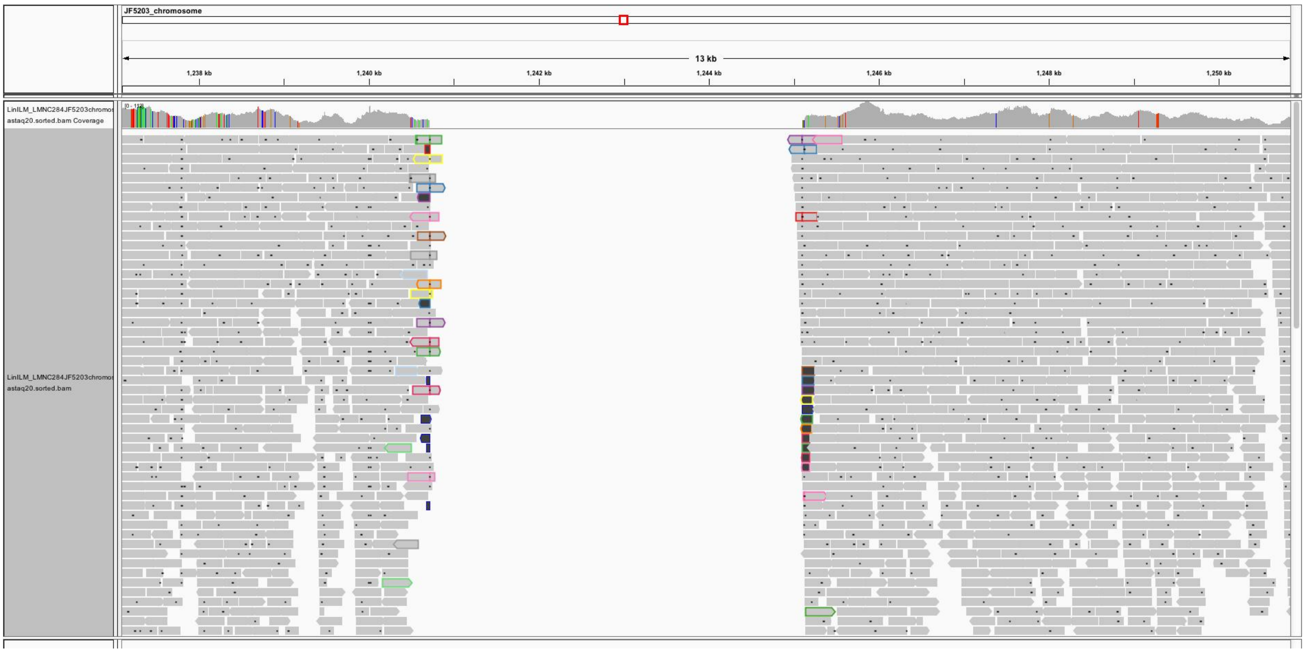

Image S8. IGV visualization of the reads of the *Listeria monocytogenes* LMNC284 strain mapped to a region of the reference genome JF5203. A deletion (absence of reads) is observed from position 1’241’370 to 1’245’059, which corresponds with the gene LMJF5203_01291 (LMOF2365_RS06250 in F2365 strain).
